# Design of financial incentive interventions to improve lifestyle behaviors and health outcomes: A systematic review

**DOI:** 10.12688/wellcomeopenres.16947.1

**Published:** 2021-06-25

**Authors:** J. Jaime Miranda, M. Amalia Pesantes, María Lazo-Porras, Jill Portocarrero, Francisco Diez-Canseco, Rodrigo M. Carrillo-Larco, Antonio Bernabe-Ortiz, Antonio J. Trujillo, Robert W. Aldridge

**Affiliations:** 1 CRONICAS Centre of Excellence in Chronic Diseases, Universidad Peruana Cayetano Heredia, Lima, 15074, Peru; 2 Department of Medicine, School of Medicine, Universidad Peruana Cayetano Heredia, Lima, 15102, Peru; 3 Division of Tropical and Humanitarian Medicine, Geneva University Hospitals & University of Geneva, Geneva, 1205, Switzerland; 4 Department of Epidemiology and Biostatistics, School of Public Health, Imperial College London, London, W2 1UA, UK; 5 Department of International Health, Johns Hopkins Bloomberg School of Public Health, Baltimore, MD 21205, USA; 6 Centre for Public Health Data Science, Institute of Health Informatics, University College London, London, NW1 2DA, UK

**Keywords:** behavioral economics, evidence synthesis, incentives, interventions, non-communicable diseases, quality, systematic review, trials

## Abstract

**Background**: Financial incentives may improve the initiation and engagement of behaviour change that reduce the negative outcomes associated with non-communicable diseases. There is still a paucity in guidelines or recommendations that help define key aspects of incentive-oriented interventions, including the type of incentive (e.g. cash rewards, vouchers), the frequency and magnitude of the incentive, and its mode of delivery.  We aimed to systematically review the literature on financial incentives that promote healthy lifestyle behaviours or improve health profiles, and focused on the methodological approach to define the incentive intervention and its delivery. The protocol was registered at PROSPERO on 26 July 2018 ( CRD42018102556).

**Methods**: We sought studies in which a financial incentive was delivered to improve a health-related lifestyle behaviour (e.g., physical activity) or a health profile (e.g., HbA1c in people with diabetes). The search (which took place on March 3 ^rd^ 2018) was conducted using OVID (MEDLINE and Embase), CINAHL and Scopus.

**Results**: The search yielded 7,575 results and 37 were included for synthesis. Of the total, 83.8% (31/37) of the studies were conducted in the US, and 40.5% (15/37) were randomised controlled trials. Only one study reported the background and rationale followed to develop the incentive and conducted a focus group to understand what sort of incentives would be acceptable for their study population. There was a degree of consistency across the studies in terms of the direction, form, certainty, and recipient of the financial incentives used, but the magnitude and immediacy of the incentives were heterogeneous.

**Conclusions**: The available literature on financial incentives to improve health-related lifestyles rarely reports on the rationale or background that defines the incentive approach, the magnitude of the incentive and other relevant details of the intervention, and the reporting of this information is essential to foster its use as potential effective interventions.

## Introduction

Many non-communicable diseases require effective engagement with lifestyle behaviours such as diet, physical activity and compliance with pharmacological medication, but this is difficult to achieve ^1^. There are major benefits to be achieved by preventive care, including both primary and secondary prevention, for example in the case of diabetes management ^2– 5^, yet adherence to healthy behaviors and pharmacological treatment remains a challenge ^6– 9^. Financial incentives may improve the initiation and engagement of behaviour change to reduce the negative outcomes associated with non-communicable diseases ^10^.

Previous systematic reviews have focused on the effect of financial incentives on lifestyle behaviours ^11– 14^, with less attention on how the financial incentives and rewards have been developed. Given the growing attention to develop incentive-oriented interventions ^10^, there is still a paucity in guidelines or recommendations that help define key aspects of such interventions, including the type of incentive (e.g. cash rewards, vouchers), the frequency and magnitude of the incentive, and its mode of delivery. To address this gap in the evidence-base, this study aimed to systematically review the literature on financial incentives to promote healthy lifestyle behaviours (e.g., physical activity) or to improve health profiles (e.g., HbA1c levels) with a specific interest in the methodological approach used to identify how the incentive intervention was defined, as well as its mechanisms of delivery.

## Methods

### Study design

A systematic review of the literature was conducted following the PRISMA guidelines ^15^ (see *Reporting guidelines* ^16^). In addition, the protocol was registered at PROSPERO on 26 July 2018 ( CRD42018102556) ^17^. We included studies that: i) had adults subjects (18+ years); ii) included financial incentives as a broad topic, i.e., no specific types of incentives (e.g. cash transfers or vouchers) were sought; and iii) the outcome of interest was a health-related lifestyle behaviour (e.g., physical activity) or a risk factor for a cardiovascular or metabolic disease (e.g., HbA1C in diabetes patients). To ensure a broad range of included studies, our review focused on the methods of the included study, rather than on the effect of the financial incentive. We also did not require a specific comparator group for the interventions. Therefore, the search approach and the selection and extraction process did not focus on effects estimates, but on the methods, including for example: criteria used to define the incentive, criteria used to define the magnitude of the incentive, and criteria used to define the recipient of the incentive ^18^.

### Search

The search was conducted in OVID (MEDLINE and Embase), Cumulative Index to Nursing and Allied Health Literature (CINAHL), and Scopus from inception to March 3 ^rd^ 2018. No language or article type restrictions were set. The search terms used in OVID are presented in *Extended data* ^16^.

### Study selection

Search results were downloaded and saved in EndNote X7 (Clarivate, Philadelphia, PA, USA). Duplicates were eliminated from the search results using EndNote duplicate reference identification. Titles and abstracts were independently screened by two reviewers; discrepancies between them were solved by a third party independently. Titles and abstracts selected from this first stage were sought in full-text, and these were studied by two reviewers independently; as before, discrepancies were solved by a third reviewer independently. These selection processes were conducted using the Rayyan online software ^19^.

### Data extraction

With the list of selected studies, information was extracted by one reviewer using a pre-specified data extraction form. Data extraction focused on items described in a framework used to document the complexity of financial incentive interventions to change health behaviours created by Adams *et al.* ^18^. In particular, the extraction form collated information about the study design, the methodological approach of the intervention, and aspects of the financial reward used, including its direction, form (e.g. cash, vouchers etc), certainty (e.g. certain if they did something, a chance of getting something), frequency, immediacy, schedule, and recipient. The details of the reviewed studies and all data extracted during the review are available at Table 1 and Table 2 (see *Underlying data* ^16^).

### Analysis

Our review aimed to describe selected characteristics of intervention studies which used economic incentives to promote healthy lifestyle behaviours as per the framework described by Adams *et al.* ^18^. To achieve this aim we undertook a narrative synthesis of the included studies and aimed to summarise the methods followed to decide upon financial incentives, magnitude of the incentives, recipient of the incentives, among other features of the economic incentives intervention. We pre-specified that we would not undertake a comprehensive analysis of the studies’ results (e.g., risk or impact estimates), including a meta-analysis, as was not the focus of our study. We also decided not to undertake a risk of bias assessment for the same reasons that this was not considered necessary for our analytical aims.

## Results

### General characteristics

**Figure 1.**
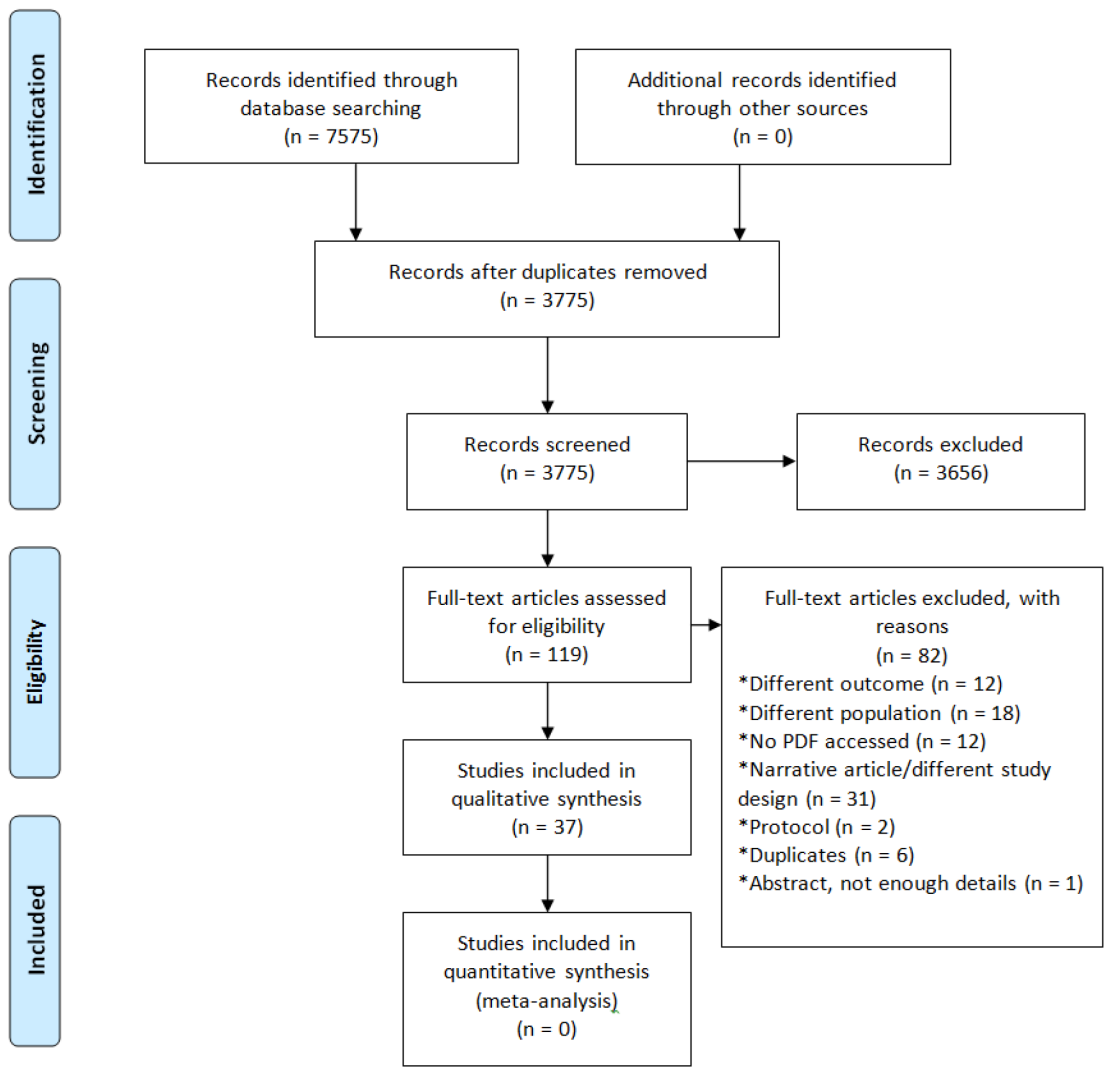
Flow diagram with the number of studies reviewed at each stage.

The search yielded 7,575 results, of which 3,656 were excluded after the initial screening. From 119 texts studied in detail, 37 were included ( Figure 1 summarizes the study selection process). All but two of the retrieved studies were conducted in high-income countries, with a majority in the USA (83.8%; 31/37), and the exception were two studies from Mexico. 40.5% (14/37) of the studies were randomized controlled trials and 26 (70.2%; 26/37) included more than 100 people.

Table 1 (see *Underlying data* ^16^) summarises the details for each included study. There were a wide range of study populations including employees, families with school aged children, and low-income families. Participants had a range of different health conditions or risk factors, such as diabetes and hypertension. Interventions targeting lifestyle behaviors and health outcomes were also equally diverse ranging from group meetings with rewards, information feedback from clinicians in the form of written letters, and process and outcome-based incentives.

### Defining the rationale for the financial incentive used

Only one study described a detailed rationale for the financial incentive used ^20^, and their methods involved conducting focus groups to understand what sort of incentives would be acceptable for their study population. None of the other included studies reported a detailed methodology or rationale to define the magnitude of the incentive, i.e. why they gave that amount of money or reward. In one of the retrieved studies, Kranker *et al.* ^21^ described that the magnitude of the incentive for one of the target goals was small because they considered such a goal (medication adherence) to be easy to achieve; in addition, they reported that “Incentive payments were moderately sized for two reasons. First, the research team needed to guarantee that total program costs would fall under a budget ceiling. Second, the research team was primarily interested in studying the effects of moderate financial incentives paired with aggressive behavior goals”.

### A review of the framework used for financial incentives

Table 2 (see *Underlying data* ^16^) summarises the financial incentives framework used for each included study. The majority of included studies (83.8%; 31/37) used positive gains for the financial rewards, with three studies using the avoidance of a negative loss and three using a mixture of rewards. Cash or vouchers exchangeable for a range of goods or services were used as the financial incentive in 83.8% (31/37) of studies and five used specific goods or services, for example, health insurance discounts or diabetes test strips (see ‘Form of reward’, Table 2, *Underlying data* ^16^). The majority of included studies 81.1% (30/37) used financial incentives that participants were certain to receive if they completed the required activity, five studies used a mixed approach (e.g. some activities were certain, but others were based upon chance), and one study used chance alone (see ‘Certainty’, Table 2, *Underlying data* ^16^). In the majority of studies the recipient of the financial incentive was the individual study participant (83.8%; 31/37) with three studies providing a mixture of individual and group rewards, and a further three studies proving household-based incentives (see ‘Recipient’, Table 2, *Underlying data* ^16^).

The magnitude of the incentive varied widely from $10 up to and including $3000. 19/37 studies rewarded some behaviours, with the remaining 18 studies rewarded all incentivised behaviours. Just under half of the studies provided financial incentives immediately after an incentivized behaviour was undertaken (45.9%; 17/37). The remaining studies delayed incentives with a maximum lag of one year after the intervention. Just over half (51.4%; 19/37) of studies used a fixed schedule for the financial incentives and 17 studies (45.9%; 17/37) used a variable schedule, although these were not always provided incrementally.

## Discussion

This work aimed to systematically review the literature on financial incentives with a specific focus on the methodological approaches followed to define the incentive intervention, rather than on the effect of the intervention. In so doing, we build upon previous systematic reviews which have focused on the effect of financial incentives on lifestyle behaviours ^11– 14^, however less attention has been placed on how the financial incentives and rewards were developed. We found that whilst being key to achieve the expected results in any given direction, little attention has been paid to a critical aspect of how to design and define the best possible incentive strategies. The average or range earned per participant was often not described and whilst targets or outcomes were described, the achievement of the tasks depends on its difficulty. If this were to be compared to a pharmacological intervention, the field of financial incentives lacks substantial attention to the design of the pharmacodynamics of the drug —minimum and maximum doses, the most effective delivery modes to guarantee higher adoption, and the frequency and spanning of the doses to be given. Experimental data indicates that insufficient incentives may paradoxically produce less motivation to engage with a given habit than if there were no incentive at all ^22^. Despite the lack of studies reporting the methodological approaches taken to define the incentive intervention, there was still a degree of consistency across the studies in terms of the direction, form, certainty, and recipient of the financial incentives used, but the magnitude and immediacy of the incentives were more varied.

These findings are relevant to inform checklists and other recommendations to improve the reporting of interventions and other endeavours using financial incentives. Either on the main manuscript, a published protocol, or on an accompanying scientific report, future studies should report on the rationale used to derive key elements of the financial incentives. More detailed information about the interventions that utilize financial incentives, an objective that adds to the broader objectives of improving the quality of the reporting of health research, is needed to critically appraise the interventions, to inform the development and testing of new interventions, and to facilitate the implementation of these interventions at a larger scale, e.g., as a public health policy. We do not have any reasons to think that the available literature did not thoroughly consider the rationale of the intervention while planning the work, yet our findings suggest that this process needs to be better and more frequently reported. The development of standardised reporting guidelines such as those used to report complex interventions (TIDieR) ^23^ or trials (CONSORT) ^24^, observational studies (STROBE) ^25^ or data linkage studies (RECORD) ^26^ should be developed in order to support this process.

Our study followed standard systematic review methods including a comprehensive search in four international data sources, but there were limitations to our approach. First, because of the aims and scope of our work we did not extract and report on the effect estimates of the economic incentives interventions. Second, we did not conduct a risk of bias assessment because we aimed to summarize the methodology of the financial incentives rather than to evaluate the quality of the whole literature. Third, we did not search bibliometric libraries specific for economic or behavioral sciences and publication bias is likely to be present. We argue that, although this strategy could have retrieved additional results, they would have focused on other aspects of the economic incentives, yet they would not report on interventions, trials, or controlled experiments with human beings, aimed at improving health outcomes.

Our study has systematically reviewed the literature on financial incentives to promote healthy lifestyle behaviours and examined the methodological approach to identify how the incentive intervention was defined and its mechanisms of delivery. We found that studies rarely report on the rationale or background to define the incentive approach, the magnitude of the incentive and other relevant details of the intervention. Future studies to guide interventions and generate evidence about the implementation of financial incentive interventions are required to fill this evidence gap.

## Data availability

### Underlying data

Figshare: Design of financial incentive interventions to improve lifestyle behaviors and health outcomes: A systematic review. https://doi.org/10.6084/m9.figshare.14659176.v3 ^16^.

This project contains the following underlying data:

- 1 Underlying data – Table 1.docx (characteristics of studies included in the review).

- 2 Underlying data – Table 2.docx (financial incentives framework summary).

### Extended data

Figshare: Design of financial incentive interventions to improve lifestyle behaviors and health outcomes: A systematic review. https://doi.org/10.6084/m9.figshare.14659176.v3 ^16^.

This project contains the following extended data:

- 3 Extended data – Search strategy.docx (Search terms as used in OVID - MEDLINE and Embase)

### Reporting guidelines

Figshare: PRISMA checklist for ‘Design of financial incentive interventions to improve lifestyle behaviors and health outcomes: A systematic review’. https://doi.org/10.6084/m9.figshare.14659176.v3 ^16^.

Data are available under the terms of the Creative Commons Attribution 4.0 International license (CC-BY 4.0).
