# Supplementary material for: Design of financial incentive interventions to improve lifestyle behaviors and health outcomes: A systematic review
Source: Wellcome Open Res. 2021 Sep 2;6:163. Originally published 2021 Jun 25. [Version 2] doi: 10.12688/wellcomeopenres.16947.2 (PMC8447049; doi:10.12688/wellcomeopenres.16947.2)
Supplement: Peer review history

# Peer review: Reviewer response for version 2

**DOI:** 10.21956/wellcomeopenres.18972.r45725

**Published:** 2021-09-16

**Authors:** Alfredo Palacios

**Affiliations:** 1 Department of Health Technology Assessment and Health Economics, Institute for Clinical Effectiveness and Health Policy (IECS), Buenos Aires, Argentina; 2 Department of Economics, Universidad de Buenos Aires, Buenos Aires, Argentina

The authors responded to my comments to version 1 of the article. I have no additional comments to version 2.

Are the rationale for, and objectives of, the Systematic Review clearly stated?

Yes

Is the statistical analysis and its interpretation appropriate?

Yes

Are sufficient details of the methods and analysis provided to allow replication by others?

Yes

Are the conclusions drawn adequately supported by the results presented in the review?

Yes

Reviewer Expertise:

Economics, Health Economics, Health Econometrics, Economic evaluation, Impact evaluation of health policies.

I confirm that I have read this submission and believe that I have an appropriate level of expertise to confirm that it is of an acceptable scientific standard.

# Peer review: Reviewer response for version 2

**DOI:** 10.21956/wellcomeopenres.18972.r45726

**Published:** 2021-09-07

**Authors:** Gustavo Angeles

**Affiliations:** 1 Department of Maternal and Child Health, University of North Carolina at Chapel Hill, Chapel Hill, NC, USA

No further comments.

Are the rationale for, and objectives of, the Systematic Review clearly stated?

Yes

Is the statistical analysis and its interpretation appropriate?

Yes

Are sufficient details of the methods and analysis provided to allow replication by others?

Yes

Are the conclusions drawn adequately supported by the results presented in the review?

Yes

Reviewer Expertise:

Health program evaluation.

I confirm that I have read this submission and believe that I have an appropriate level of expertise to confirm that it is of an acceptable scientific standard.

# Peer review: Reviewer response for version 1

**DOI:** 10.21956/wellcomeopenres.18701.r44671

**Published:** 2021-08-16

**Authors:** Gustavo Angeles

**Affiliations:** 1 Department of Maternal and Child Health, University of North Carolina at Chapel Hill, Chapel Hill, NC, USA

This is a valuable paper. The topic of the systematic review is very relevant as it identifies the magnitude of an important gap in the reporting of studies of financial incentives for modifying health behaviors. In my view, the results of this review ought to be disseminated and standards/guidelines of reporting should indicate the need for including information on key design features of the incentive being examined. 

 A drawback of this review is that it is not clear why papers from the economic and development literature were omitted. The rationale for that omission is not clear to me. Other than that, this is a valuable review.

Are the rationale for, and objectives of, the Systematic Review clearly stated?

Yes

Is the statistical analysis and its interpretation appropriate?

Yes

Are sufficient details of the methods and analysis provided to allow replication by others?

Yes

Are the conclusions drawn adequately supported by the results presented in the review?

Yes

Reviewer Expertise:

Health program evaluation.

I confirm that I have read this submission and believe that I have an appropriate level of expertise to confirm that it is of an acceptable scientific standard.

# Response

**Published:** 2021-08-21

**Authors:** Jaime Miranda

**Affiliations:** Universidad Peruana Cayetano Heredia, Peru

We are grateful for the feedback provided. The comment that "the results of this review ought to be disseminated and standards/guidelines of reporting should indicate the need for including information on key design features of the incentive being examined" is precisely the key motivation for this work.

 We agree with the reviewer that an important limitation of our study was that our search strategy did not include databases for the fields of economics and development. This has been highlighted as a limitation in the revised version of our work.

 Thank you for your time and feedback.

# Peer review: Reviewer response for version 1

**DOI:** 10.21956/wellcomeopenres.18701.r44674

**Published:** 2021-08-03

**Authors:** Alfredo Palacios

**Affiliations:** 1 Department of Health Technology Assessment and Health Economics, Institute for Clinical Effectiveness and Health Policy (IECS), Buenos Aires, Argentina; 2 Department of Economics, Universidad de Buenos Aires, Buenos Aires, Argentina

The article addresses a topic of great relevance for the design and implementation of financial interventions to improve the population’s behaviours and lifestyles. The study contributes to understanding the key characteristics of these financial interventions and how the applied studies designed (or not) such financial incentives.

 After reviewing the article, I have three main comments and one minor comment.

**Major comments:**

1. The authors carried out the bibliographic search in March 2018 (over three years ago). In a subject that has had a considerable dynamism, I consider three years is a long time for a systematic review. I suggest the authors update the bibliographic search. If this is not possible because of the workload that it could imply, the authors should establish this point as a study limitation.
2. The authors searched for published articles only in biomedical repositories (such as MEDLINE). From my point of view, this represents the most important limitation of the study, given the economic nature of the subject and considering that in later years the economic literature (in fields such as Behavioral Economics and Economic Development) has produced several applied studies in the topic. Although the authors mention this as a study limitation, I assume they relativize it, suggesting that economics journals do not focus on this type of applied studies. I consider that this is not correct, and the authors should revalue the relevance of this limitation.
3. The review focus on the empirical aspects of the design of financial incentives, but I consider the authors could enhance the article if they present or reference (in the introduction and/or discussion section) a conceptual framework from Behavioral Economics on individual behaviours in the face of financial incentives. Concepts such as loss aversion, overweighting of small probabilities, hyperbolic discounting, increasing payoffs and reference points have ceased to be characteristic of economics, spreading to the field of public health. Some studies considering this are:
  Vlaev, Ivo, *et al.* “Changing health behaviours using financial incentives: a review from behavioural economics.” BMC public health 19.1 (2019): 1-9 ^1^.
  Mitchell, Marc S., *et al.* “Financial incentives for physical activity in adults: systematic review and meta-analysis.” British Journal of Sports Medicine 54.21 (2020): 1259-1268 ^2^.

**Minor comment:**

1. The citations/references of the 37 studies included in the qualitative synthesis are not available either in the manuscript or in the tables. The authors should resolve this.

Are the rationale for, and objectives of, the Systematic Review clearly stated?

Yes

Is the statistical analysis and its interpretation appropriate?

Yes

Are sufficient details of the methods and analysis provided to allow replication by others?

Yes

Are the conclusions drawn adequately supported by the results presented in the review?

Yes

Reviewer Expertise:

Economics, Health Economics, Health Econometrics, Economic evaluation, Impact evaluation of health policies.

I confirm that I have read this submission and believe that I have an appropriate level of expertise to confirm that it is of an acceptable scientific standard, however I have significant reservations, as outlined above.

## References

[1] : Changing health behaviors using financial incentives: a review from behavioral economics.*BMC Public Health*.2019;19(1) : 10.1186/s12889-019-7407-8105910.1186/s12889-019-7407-831391010PMC6686221

[2] : Financial incentives for physical activity in adults: systematic review and meta-analysis.*Br J Sports Med*.2020;54(21) : 10.1136/bjsports-2019-1006331259-126810.1136/bjsports-2019-10063331092399

# Response

**Published:** 2021-08-21

**Authors:** Jaime Miranda

**Affiliations:** Universidad Peruana Cayetano Heredia, Peru

We are grateful for the constructive comments provided by the reviewer. We appreciate that our work was deemed “a topic of great relevance for the design and implementation of financial interventions to improve the population’s behaviours and lifestyles.” Here we proceed to respond to his comments.

**Comment #1**: The authors carried out the bibliographic search in March 2018 (over three years ago). In a subject that has had a considerable dynamism, I consider three years is a long time for a systematic review. I suggest the authors update the bibliographic search. If this is not possible because of the workload that it could imply, the authors should establish this point as a study limitation.

**Response #1**: Yes, this is a limitation and we have added it in our revised version. The new text reads:

*“Finally, our search was conducted up to 2018, an inherent limitation given the considerable dynamism in the field. Yet, this does not preclude the recommendation that guidelines for the reporting of incentive-related studies are needed, in particular the need for including information on key design features of the incentive being examined.”*

**Comment #2**: The authors searched for published articles only in biomedical repositories (such as MEDLINE). From my point of view, this represents the most important limitation of the study, given the economic nature of the subject and considering that in later years the economic literature (in fields such as Behavioral Economics and Economic Development) has produced several applied studies in the topic. Although the authors mention this as a study limitation, I assume they relativize it, suggesting that economics journals do not focus on this type of applied studies. I consider that this is not correct, and the authors should revalue the relevance of this limitation.

**Response #2**: Point taken. We feel partly reassured that the databases used for our review are key ones for the field of health economics, as per https://guides.himmelfarb.gwu.edu/HealthEconomics/bibliographic-databases. Another reassurance is that there will be some degree of overlap between economic and health databases as exemplified by this publication that appears on IDEAS database ( https://ideas.repec.org/a/eee/ehbiol/v41y2021ics1570677x2100006x.html) and PubMed/MEDLINE ( https://pubmed.ncbi.nlm.nih.gov/33611235/). Yet, we concur that not exploring economics and development literature remains a limitation, and we have reworded that limitation in light of the feedback provided.

**Comment #3**: The review focus on the empirical aspects of the design of financial incentives, but I consider the authors could enhance the article if they present or reference (in the introduction and/or discussion section) a conceptual framework from Behavioral Economics on individual behaviours in the face of financial incentives. Concepts such as loss aversion, overweighting of small probabilities, hyperbolic discounting, increasing payoffs and reference points have ceased to be characteristic of economics, spreading to the field of public health. Some studies considering this are:

**Response #3**: Thank you, this is very useful and we have added the two suggested references as well as others. As suggested, we have expanded about this in the introduction and the discussion **.** The new text reads:

*“The field of behavioural economics has provided critical insights to influence public policy [REF], and incentives are one of many tools available. Concepts such as present bias, loss aversion, choice overload, and reference points, among others, have now spread to the field of public health and healthcare delivery [REF].”*

*“Different scheme designs are likely to lead to different outcomes, and hence, it is important to think about the most effective design of incentive schemes [REF], as subtle design choices in how incentives are situated, framed, or deployed can have substantial effects on their success [REF].”*

**Comment #4 (minor)**: The citations/references of the 37 studies included in the qualitative synthesis are not available either in the manuscript or in the tables. The authors should resolve this.

**Response #4**: The tables (Underlying data, available at http://www.doi.org/10.6084/m9.figshare.14659176.v3) provide the first author and the year of publication, together with additional details of the study. We have added the full citation to Table 1 (underlying data).
